# Socioeconomic inequalities in parent-reported and teacher-reported psychological well-being

**DOI:** 10.1136/archdischild-2014-306288

**Published:** 2014-08-27

**Authors:** Hannah Lewis, Steven Hope, Anna Pearce

**Affiliations:** 1 Great Ormond Street Hospital, London, UK; 2 Population, Policy and Practice Programme, UCL Institute of Child Health, London, UK

**Keywords:** Socio-economic factors, Child Psychology, Cohort studies, Parent-teacher reports

## Abstract

**Objective:**

To determine whether there are differences in the social gradient of parent-reported and teacher-reported child psychological well-being.

**Design:**

Secondary data analysis comparing ratings of child psychological well-being (Strengths and Difficulties Questionnaire, SDQ) in the UK Millennium Cohort Study at 7 years by socioeconomic circumstances (SEC). A number of measures of SEC were tested; results are reported for maternal education. From a sample of 13 168 singletons who participated at the age of 7 years, complete data were available for 8207 children.

**Results:**

There was a social gradient in SDQ scores reported by parents and teachers, with ‘borderline/abnormal’ scores more prevalent in children with lower-educated mothers. However, the gradient was more marked in parent report compared with teacher report, and discrepancies between parent and teacher reports were greatest for children from higher SECs.

**Conclusions:**

The social gradient in child psychological well-being, although present, was weaker in teacher report compared with parent report. This may be because children behave differently in school and home settings, or parents and teachers demonstrate reporting bias.

**What is already known on this topic?**

- It has been widely demonstrated that children from more disadvantaged backgrounds have poorer psychological well-being than their more advantaged peers.
- However, the majority of research on younger children's psychological well-being uses single informants, predominantly a parent.

**What this study adds?**

- We examined inequalities (by maternal education, and repeated for social class, lone parenthood and tenure) in borderline/abnormal scores on the Strengths and Difficulties Questionnaire, as reported by parents and teachers.
- While there was a strong social gradient in scores from both informants, it was significantly stronger when reported by parents.
- Future research should explore the extent to which these differences are explained by children behaving differently in school and home settings or reporting bias.

## Introduction

The association between socioeconomic disadvantage and poor psychological well-being in young children is well established, but is largely based on parental report.^1^ Assessments from informants who see children in other settings may provide further insights. Primary school teachers, who spend long periods with pupils, watching them interact with peers and other adults, are the most frequently used additional informants in research. However, inequalities in psychological well-being, as rated by parents and teachers, have rarely been compared in young children. We aimed to do this using data from a large contemporary UK cohort.

## Method

### Subjects and design

We examined data from the Millennium Cohort Study (MCS), a longitudinal study of children born in the UK between 2000 and 2002 (http://www.cls.ioe.ac.uk/mcs). Surveys were carried out with the main respondent (97% were natural mothers). Thirteen thousand one hundred and sixty-eight singleton children at the age of 7 years took part in the survey in which the child's primary school teacher was also invited to complete a survey. Data were obtained from the UK Data Archive, University of Essex in May 2012.

Child psychological well-being was assessed using the Strengths and Difficulties Questionnaire (SDQ)^2^ which was completed by the main respondent (by computer) and the child's teacher (by mail). We used the total difficulties score, which is the sum of four difficulties components (peer problems, conduct disorders, hyperactivity and emotional problems) and ranges from 0 to 40 (a higher score indicates more difficulties). Children were classified using recommended cut-offs^3^ as having ‘borderline/abnormal’ scores (>13 parent; >11 teacher). We ran sensitivity analyses with the continuous score.

Two thousand seven hundred and thirty-four (20.0%) children were missing a parent-reported and 5394 (39.4%) a teacher-reported SDQ score, largely due to missing entries for just one or two items on one or more of the SDQ components. In these circumstances, we rescaled the average using the other completed items.^3^ This reduced missing data to 3.6% for parents although it remained high (36.9%) for teachers. Eight thousand four hundred and forty-three (61.7%) children had total difficulties scores from both informants. It has been demonstrated elsewhere that after accounting for the survey design and child non-response, the teacher data are close to being missing at random.^4^ Nevertheless, we multiply imputed data as a sensitivity analysis. As expected, findings were very similar; therefore, complete case findings are presented.

We examined the mother's highest educational qualification achieved by the time the child was 7 years of age, classified as: ‘degree’, ‘diploma’, ‘A levels’, ‘GCSE A*-C’, ‘GCSE D-G’ or ‘none’. Two hundred and thirty-six children were missing education data, either because the mother had not reported it or because she reported ‘other qualifications’ (excluded because it was not possible to determine the level of qualification). Additional measures of socioeconomic circumstances (SEC) were explored, representing different aspects of disadvantage: income poverty, lone parenthood, employment-based social class and housing tenure. Since a similar pattern of results was found across SECs, only those by maternal education are reported.

### Statistical analysis

The working sample comprised 8207 children with SDQ reports and maternal education data. All analyses were conducted in Stata/SE 12 (Stata Corporation, Texas, USA) using ‘svy’ commands to allow for clustered sampling design and attrition up to the age of 7 years. Five thousand and forty-four teachers took part in the survey, providing SDQ scores for a mean 1.6 MCS children (modal value: 1). The clustering of children within teachers arose due to the sample design, and is therefore accounted for by the survey weights.^4^

We estimated prevalence of ‘borderline/abnormal’ SDQ by maternal education. Risk ratios (RR) and 95% CI for ‘borderline/abnormal’ scores were estimated using Poisson regression for parent and teacher reports separately, according to maternal education. Next, we examined whether the social gradient in SDQ scores varied by informant, through testing for an informant–education interaction. As a sensitivity analysis, we repeated analyses with continuous total difficulties scores using linear regression. In multinomial logistic regression models, we then estimated Relative Risk Ratios (RRR) (according to maternal education) for the following combinations of SDQ outcomes: rated ‘normal’ by both informants, rated ‘normal’ by parent and ‘borderline/abnormal’ by teacher, rated ‘borderline/abnormal’ by teacher and ‘normal’ by parent, and rated ‘borderline/abnormal’ by both informants. Inequalities did not vary by gender for parent or teacher reports, when examined with ‘borderline/abnormal’ cut-offs or mean scores (p>0.1).

## Results

In general, the prevalence of ‘borderline/abnormal’ total difficulty scores for both informants increased incrementally as maternal education decreased, with the exception of the diploma category, with highest risks of ‘borderline/abnormal’ scores among children whose mothers had no qualifications and lowest risks for those with degrees (table 1). A significant interaction between informant and education indicated that inequalities in ‘borderline/abnormal’ scores were greater when reported by a parent than a teacher. For example, compared with children whose mothers had no qualifications, those whose mothers had a degree were four times less likely to display ‘borderline/abnormal’ behaviour when rated by a parent (RR 0.3 (CI 0.2 to 0.3)), but only half as likely to have a ‘borderline/abnormal’ score when rated by a teacher (RR=0.4 (CI 0.4 to 0.5)). The pattern was similar for mean scores (data not shown). Informant differences were more apparent among children of more educated mothers: the prevalence of ‘borderline/abnormal’ behaviour in children whose mothers had no qualifications were similar for parent and teacher reports (24.2% and 25.5%, respectively); by contrast, prevalence differed in children whose mothers had a degree (6.0% and 11.1%).

**Table 1:** Weighted percentages (N) and risk ratios (CIs) for parent-reported and teacher-reported ‘borderline/abnormal’ Strengths and Difficulties Questionnaire (SDQ) scores, according to maternal education

	% (N) All	% (N) ‘borderline/abnormal’ score		RRs (CIs) ‘borderline/abnormal’ score	
		Parent rated	Teacher rated	Parent rated	Teacher rated
None	16.6 (1687)	24.2 (260)	25.5 (295)	–	–
GCSE D-G	10.1 (856)	20.7 (160)	24.3 (193)	0.9 (0.7 to 1.1)	1.0 (0.8 to 1.2)
GCSE A*-C	9.9 (882)	12.9 (346)	16.2 (453)	0.5 (0.4 to 0.7)	0.6 (0.5 to 0.8)
A Levels	36.0 (2833)	9.1 (79)	12.9 (107)	0.4 (0.3 to 0.5)	0.5 (0.4 to 0.6)
Diploma	11.0 (828)	10.1 (80)	14.7 (128)	0.4 (0.3 to 0.5)	0.6 (0.5 to 0.7)
Degree	14.3 (1121)	6.0 (91)	11.1 (117)	0.3 (0.2 to 0.3)	0.4 (0.4 to 0.5)
Total	100 (8207)	13.8 (1073)	17.2 (1410)		

RR, risk ratio.

**Figure 1:**
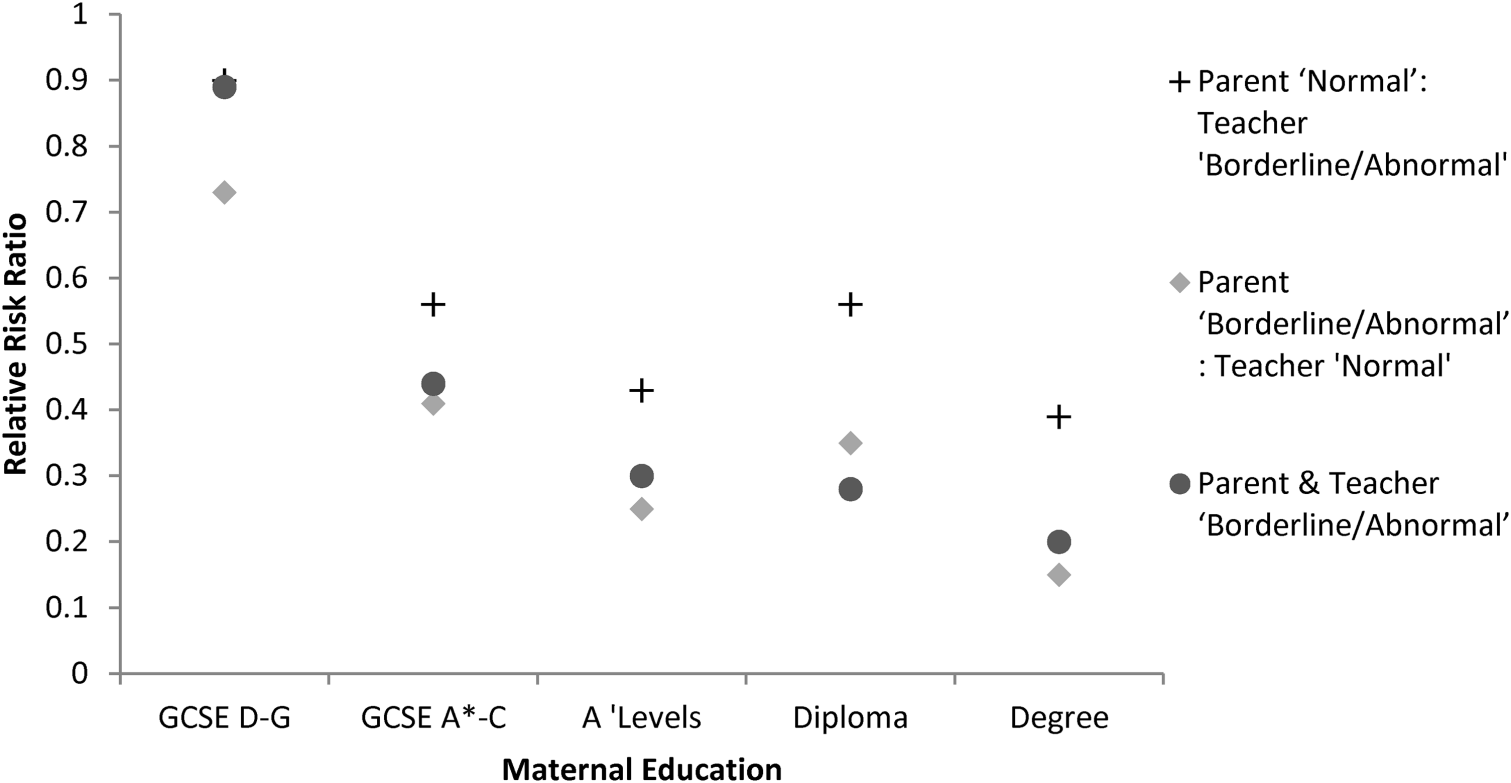
Relative risk ratios (see table 2 for the CIs of relative risk ratios) for combined parent-reported and teacher-reported Strengths and Difficulties Questionnaire scores according to maternal education.

Combinations of parent and teacher reports were then examined in a multinomial model, with children rated as ‘normal’ by parent and teacher as the baseline group. Children with more qualified mothers were less likely to be rated ‘borderline/abnormal’ by one or both informants than those whose mothers had no qualifications (table 2). The social gradient was strongest when a child was rated ‘borderline/abnormal’ by both informants or rated ‘normal’ by a teacher and ‘borderline/abnormal’ by a parent (figure 1); this latter finding might be expected given the stronger social gradient in parent-reported SDQ (table 1).

**Table 2:** Relative risk ratios (RRR) (95% CIs) for combined parent-reported and teacher-reported Strengths and Difficulties Questionnaire (SDQ) scores, according to maternal education

Parent SDQ Score	‘Normal’	‘Normal’	‘Borderline/abnormal’	‘Borderline/abnormal’
Teacher SDQ Score	‘Normal’	‘Borderline/abnormal’	‘Normal’	‘Borderline/abnormal’
Total weighted % and N				
	76.4 (6448)	10.9 (922)	6.9 (585)	5.8 (488)
Weighted % (N) by maternal education				
* *None	61.0 (685)	14.8 (176)	13.6 (141)	10.7 (119)
GCSE D-G	65.2 (556)	14.1 (112)	10.5 (79)	10.2 (81)
GCSE A*-C	76.8 (2190)	10.3 (297)	7.0 (190)	5.9 (156)
A Levels	82.4 (731)	8.5 (72)	4.8 (44)	4.3 (35)
Diploma	79.1 (682)	10.8 (94)	6.2 (46)	3.9 (34)
Degree	86.0 (1460)	8.1 (136)	3.0 (50)	3.0 (41)
RRR (95% CI) by maternal education				
None	–	–	–	–
GCSE D-G	–	0.9 (0.7 to 1.2)	0.7 (0.5 to 1.1)	0.9 (0.6 to 1.3)
GCSE A*-C	–	0.6 (0.4 to 0.7)	0.4 (0.3 to 0.6)	0.4 (0.3 to 0.6)
A Levels	–	0.4 (0.3 to 0.6)	0.3 (0.2 to 0.4)	0.3 (0.2 to 0.5)
Diploma	–	0.6 (0.4 to 0.8)	0.4 (0.2 to 0.6)	0.3 (0.2 to 0.4)
* *Degree	–	0.4 (0.3 to 0.5)	0.2 (0.1 to 0.2)	0.2 (0.1 to 0.3)

## Discussion

Previous research has identified a social gradient in young children's psychological well-being when reported by parents. However, there has been scant research comparing socioeconomic inequality between informants. An analysis of the 1999 and 2004 British Child and Adolescent Mental Health Surveys indicated that parent-reported and teacher-reported SDQ scores (in 11-year-olds to 15-year-olds) were similarly related to socioeconomic characteristics, and more strongly than for reports by young people themselves.^5^ Our findings indicate that teachers’ assessment of young children's psychological well-being has a weaker, but still significant, relationship with children's SECs. These differences may be attributable to a number of factors related to SECs. Reporting bias could account for the lower prevalence of ‘borderline/abnormal’ behaviour in parent reports compared with teacher reports in children from more advantaged backgrounds. For example, better educated mothers may be more inclined to report their child's behaviour optimistically; or teachers may adjust their expectations for, or assessment of, behaviour according to their knowledge of the child's background. On the other hand, advantaged children may be more likely to alter their behaviour according to setting, with for example, less stringent boundaries and rules at home than at school. However, we were not able to examine these hypotheses in this dataset.

To our knowledge, this is the first study to compare the social gradient in parent and teacher reports of young children's SDQ scores in the UK, and we do this in a large, representative cohort. Similar associations were also observed for alternative measures of SECs, and when analyses were repeated using a continuous score. SDQ is a validated and reliable measure when reported by parents and teachers.^2^ ^6^ The correlation between parent and teacher SDQ scores was r=0.5 (p<0.001), which is similar or greater to that reported in other studies.^5^ ^7^ We used response weights to account for attrition, however, this cannot account for item non-response. A large proportion of children did not have teacher-reported SDQ. However, analyses from published technical data^4^ and our sensitivity analysis using multiple imputation both indicate that bias resulting from missing data is negligible.

A social gradient in SDQ scores was observed for both informants, but was stronger when reported by a parent; future studies should consider this when examining and interpreting inequalities in child psychological well-being using a single category of informant. The aim of this short report was to describe, for the first time, differences in the socioeconomic gradient in parent-reported and teacher-reported SDQ scores in a nationally representative sample. Future research might examine the mechanisms behind these differences, which could include school and teacher characteristics, parental mental health and parenting style. Finally, it will be informative to examine the extent to which parent and teacher scores predict inequalities in child psychological outcomes at later ages.
